# Supplementary material for: The Role of Oxidoreductase-Like Protein Olp1 in Sexual Reproduction and Virulence of Cryptococcus neoformans
Source: Microorganisms. 2020 Nov 4;8(11):1730. doi: 10.3390/microorganisms8111730 (PMC7694259; doi:10.3390/microorganisms8111730)
Supplement: Supplementary file 1 [file microorganisms-08-01730-s001.pdf]

Supplemental figure legends

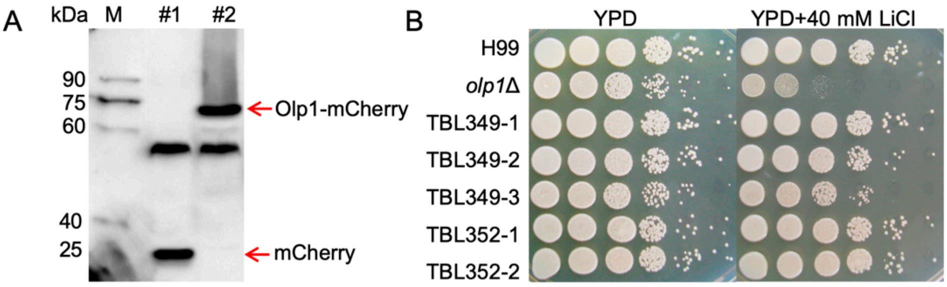

Figure S1. Verification of Olp1-mCherry strains. (A) Detection of Olp1-mCherry protein by Western blot; M: Easysee protein marker; 1: mCherry protein; 2: Olp1-mCherry fusion protein. (B) Phenotype verification of Olp1-mCherry strains; Olp1-mCherry strains could rescue the LiCl sensitive phenotype of *olp1*Δ mutants.
